# Ferritin Structure from *Mycobacterium tuberculosis*: Comparative Study with Homologues Identifies Extended C-Terminus Involved in Ferroxidase Activity

**DOI:** 10.1371/journal.pone.0018570

**Published:** 2011-04-08

**Authors:** Garima Khare, Vibha Gupta, Prachi Nangpal, Rakesh K. Gupta, Nicholas K. Sauter, Anil K. Tyagi

**Affiliations:** 1 Department of Biochemistry, University of Delhi South Campus, New Delhi, India; 2 Ram Lal Anand College, University of Delhi, New Delhi, India; 3 Physical Biosciences Division, Lawrence Berkeley National Laboratory, Berkeley, California, United States of America; Institute of Microbial Technology, India

## Abstract

Ferritins are recognized as key players in the iron storage and detoxification processes. Iron acquisition in the case of pathogenic bacteria has long been established as an important virulence mechanism. Here, we report a 3.0 Å crystal structure of a ferritin, annotated as Bacterioferritin B (BfrB), from *Mycobacterium tuberculosis* (Mtb), the causative agent of tuberculosis that continues to be one of the world's deadliest diseases. Similar to the other members of ferritin family, the Mtb BfrB subunit exhibits the characteristic fold of a four-helical bundle that possesses the ferroxidase catalytic centre. We compare the structure of Mtb BfrB with representatives of the ferritin family belonging to the archaea, eubacteria and eukarya. Unlike most other ferritins, Mtb BfrB has an extended C-terminus. To dissect the role of this extended C-terminus, truncated Mtb BfrB was purified and biochemical studies implicate this region in ferroxidase activity and iron release in addition to providing stability to the protein. Functionally important regions in a protein of known 3D-structure can be determined by estimating the degree of conservation of the amino-acid sites with its close homologues. Based on the comparative studies, we identify the slowly evolving conserved sites as well as the rapidly evolving variable sites and analyze their role in relation to structure and function of Mtb BfrB. Further, electrostatic computations demonstrate that although the electrostatic environment of catalytic residues is preserved within the family, extensive variability is exhibited by residues defining the channels and pores, in all likelihood keeping up with the diverse functions executed by these ferritins in varied environments.

## Introduction

Iron represents an essential element for almost all organisms as it is involved in many biological processes such as electron transport, DNA synthesis and various redox reactions [1]. While iron deprivation in an organism causes cessation of growth leading to its death, an increase in iron results in the production of reactive oxygen species (ROS) that damage the basic components of the cell such as nucleic acids and lipid membranes [1]. Hence, the need for maintaining iron homeostasis is imperative for the cellular growth of organisms. Ferritins are ancient proteins that store iron away from the delicate machinery of the cell to be released again in a controlled fashion at the time of need. The iron storage mechanism involves binding of ferrous iron to ferritin protein followed by migration to the ferroxidase catalytic site where ferrous iron [Fe(II)] is oxidized to the ferric [Fe(III)] state. Oxygen and hydrogen peroxide represent the major cellular oxidants for this oxidation reaction [2]. Subsequently Fe(III) is transferred and sequestered as ferric mineral in the storage cavity of ferritin making it available for the cell at the time of iron deprivation [3].

Ferritin molecules are present in all forms of life and are key contributors in maintaining iron homeostasis [4], [5]. The ferritin family consists of three sub-families, the typical iron storing ferritin (Ftn) present in both prokaryotes and eukaryotes, the heme containing bacterioferritin (Bfr) and DNA-binding protein from starved cells (Dps) found only in prokaryotes. The representative 3-dimensional crystal structures from all three ferritin subfamilies are available in a number of species, e.g., human [6], [7], frog [8], [9], [10], horse [11], [12], soybean [13], insect [14], *Escherichia coli* [15], *Campylobacter jejuni* (PDB code: 1krq; unpublished work), *Pyrococcus furiosus* [16], *Archaeoglobus fulgidus* [17], *Helicobacter pylori* [18], *Thermotoga maritima* (PDB code:1vlg; unpublished work), *Listeria innocua* [19], *Desulfovibrio desulfuricans* [20], *Bacillus brevis* [21] and others. Despite significant differences in the primary sequences, the tertiary and quaternary structures of various ferritins are found to be strikingly similar. The ferritin subunit structure, a characteristic four-helical bundle (helices A–D) plus a long loop that links helix B to helix C, essentially defines the iron storage function of the protein. The ferritin and Bfr proteins exhibit the same quaternary structure where 24 subunits assemble in octahedral 432-symmetric arrangements to form a roughly spherical protein shell enclosing a hollow cavity that holds ∼5000 molecules of Fe(III) mineral. Ferritin from hyperthermophilic archaeon *Archaeoglobus fulgidus* (PDB code: 1s3q) is an exception as the 24 subunits in this case assemble into a tetrahedral 23 symmetry [17]. The shell contains numerous pores and channels responsible for steering iron, oxidants, reductants, chelators and other small molecules in and out of the ferritin molecule [22]. Both ferritin and Bfr assembly can be composed of either homologous or heterologous subunits. The foremost difference between these two sub-families is the presence of a heme moiety at the interface between two-fold related subunits in Bfr proteins (not including *M. tuberculosis* bacterioferritin B, which is considered to be part of the ferritin subfamily). Dps, the third ferritin subfamily, is comprised of only 12 subunits organized into a 23 symmetrical tetrahedral arrangement, and being smaller in size has a lower iron-storage capacity as compared to ferritin and Bfr. This is also a reflection of the different physiological role of Dps in contrast to the primary iron-storage function of ferritin and Bfr proteins. Dps protects DNA from degradation in the presence of hydrogen peroxide and Fe(II) ions [23]. Dps uses hydrogen peroxide as an oxidant whereas ferritins utilize di-oxygen as the physiological oxidant [24]. The 24meric ferritin and Bfr proteins have an intra-subunit ferroxidase centre, whereas the 12meric Dps proteins possess an inter-subunit ferroxidase centre. Furthermore, gene expression of various ferritins appears to be differentially regulated and modulated by many environmental factors [25]. Literature on ferritins corroborates the importance of these proteins for the adaptive response of the organism to environmental changes [26], [27], [28] and the coexistence of multiple ferritin paralogues within an organism is indicative of the fact that they fulfill disparate physiological roles.

The iron acquisition and iron storage pathways of *Mycobacterium tuberculosis* (Mtb) have been linked to its growth and disease-causing ability [29], [30], [31], [32]. Therefore, the iron storage mycobacterial ferritins represent attractive targets for the development of antitubercular drugs [33], [34], [35], [36]. The Mtb genome has two ferritin paralogues [37]. The crystal structure of BfrA/Rv1876, a heme-containing bacterioferritin, has been recently reported [38]. BfrB/Rv3841, the subject of this paper, is a non-heme binding ferritin. Though both mycobacterial ferritins are upregulated when the organism is cultured in iron rich media [32], during adaptation to stationary phase and low-oxygen dormancy only BfrB is found to be abundant [36]. Interestingly, its presence in the extracellular milieu [39] suggests another role for this protein in addition to the indicated function in latency [40].

In the ferritin context, the term “BfrB” is misleading as this protein is not a member of the heme-containing Bfr subfamily. However, in accordance with the published genome annotation [37], we will continue addressing the protein as BfrB. This study describes the 3.0 Å crystal structure of Mtb BfrB. To gain further insights into the iron storage process of Mtb BfrB, the structure is analyzed with respect to available structural and functional information on representative members of the ferritin family.

## Results

### Expression and Purification of Mtb BfrB

**Figure 1:**
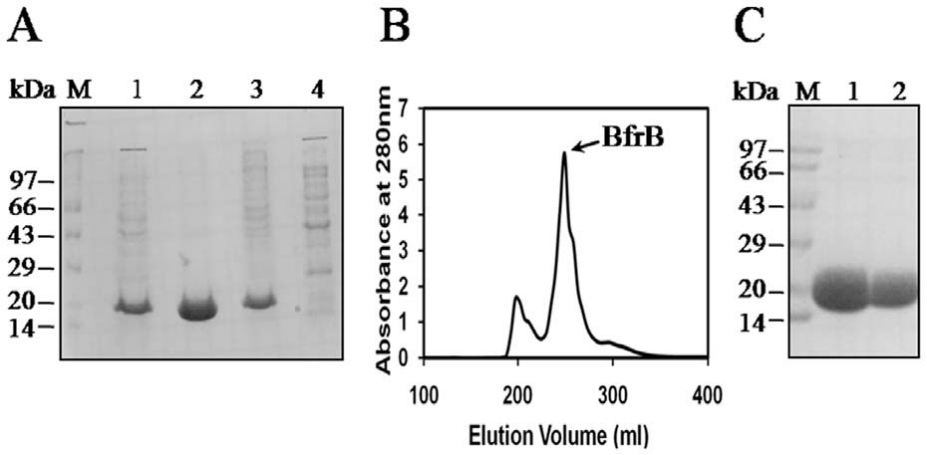
Expression and Purification of Mtb BfrB. (A) Ammonium sulfate (AS) fractionation of the cell lysate. M – Molecular weight markers, lane 1 – cell lysate, lane 2 – 0–25% AS pellet, lane 3 – 25–40% AS pellet, lane 4 – 40–50% AS pellet. (B) Elution profile of the gel-filtration chromatography of 0–25% AS fraction of BfrB using a Sephacryl S-300 column. (C) Analysis of purified BfrB on a 12.5% SDS-polyacrylamide gel. M – Molecular weight markers, lanes 1& 2 - purified BfrB.

The gene encoding Mtb BfrB (Rv3841) was cloned into plasmid pET21c and the recombinant protein was overexpressed in *E. coli*. The protein was purified to homogeneity in two steps. The first step of ammonium sulfate fractionation of the cell lysate, where ∼90% of the target protein precipitated in the 0–25% fraction (Figure 1A, lane 2) was followed by a second step of size-exclusion chromatography using a Sephacryl – S300 gel filtration column. Figure 1B shows the elution profile of the chromatography and Figure 1C demonstrates the purity of the protein on a 12.5% SDS polyacrylamide gel.

### Crystallization, Structure Determination and Refinement

Crystals of recombinant BfrB were grown at 293 K by using a starting protein concentration of 11 mg/ml from a solution of 20% PEG 3350 in 0.1 M Tris pH 8.5. The crystals diffracted up to 3.0 Å and belonged to the monoclinic C121 space group with unit cell dimensions of a = 226.1 Å, b = 226.2 Å, c = 113.7 Å and β = 94.4°. The molecular weight of BfrB being ∼20,000 Da, the Matthews constant (V_m_) of 2.9 Å^3^/Da with solvent content of 57%, suggested 24 molecules of the protein in the asymmetric unit, corresponding to an entire 432 symmetric octahedron. Molecular replacement with a homologous 24-mer search model gave a clear solution. After refinement the final *R*_work_ and *R*_free_ were 20.9% and 23.6%, respectively. Analysis of the final model using MOLPROBITY [41] shows a good stereochemistry with an rmsd of 0.009 Å for the bond lengths and 1.058° for the bond angles. Ramachandran outliers are only 0.9% with 98.5% residues in the favored region of the plot. Statistics pertaining to data collection, processing, refinement and model quality are summarized in Table 1.

**Table 1:** Data collection and refinement statistics for Mtb BfrB crystals.

Diffraction data	
Space group	C121
Unit-cell parameters	a = 226.1 Å, b = 226.2 Å, c = 113.7 Å, β = 94.4°
Temperature (K)	120
Wavelength (Å)	1.5418
Crystal-to detector distance (mm)	180
Resolution limit (Å)	31.9–3.0 (3.1–3.0)
No. of observed reflections	226119 (30062)
No. of Unique reflections	92122 (13564)
Completeness (%)	81.4 (82.2)
Average redundancy	2.5 (2.2)
Mean I/( (I)	7.8 (3.1)
†Rmerge (%)	13.7 (25.8)
No. of molecules in ASU	24
Matthews coefficient (Å^3^ Da^−1^)	2.9
Solvent content (%)	57

Refinement and model quality	
Resolution (Å)	31.9–3.0 (3.08–3.00)
No. of reflections used	92083 (6492)
Completeness (%)	81.2 (82)
‡R_work_/§R_free_ (%)	20.9/23.6 (26.5/31.2)
rms deviation bond lengths (Å)	0.009
rms deviation bond angles (°)	1.058
Average B factor (Å^2^)	16.1
Number of protein/solvent atoms	31623/360
Ramachandran validation	
Residues in favoured regions (%)	98.5
Residues in allowed regions (%)	0.6
Residues in disallowed regions (%)	0.9

Values in parentheses are for the highest resolution shell.

† Rmerge = ∑hkl∑i |Ihkl−<Ihkl>|/∑hkl∑iIhkl, where Ihkl is the intensity of an individual measurement of the reflection with Miller indices *h*, *k* and *l* and <*I_hkl_*> is the mean intensity of redundant measurements of that reflection.

‡ *R*_work_ = Σ*_hkl_* |*Fo_(hkl)_*−*Fc_(hkl)_*|/Σ*_hkl_* |*Fo_(hkl)_*|, where *Fo* and *Fc* are observed and calculated structure factors, respectively.

§ *R*_free_ calculated for a randomly selected subset of reflections (10%) that were omitted during the refinement.

### Mtb BfrB contains an uncommon extended C-terminus

**Figure 2:**
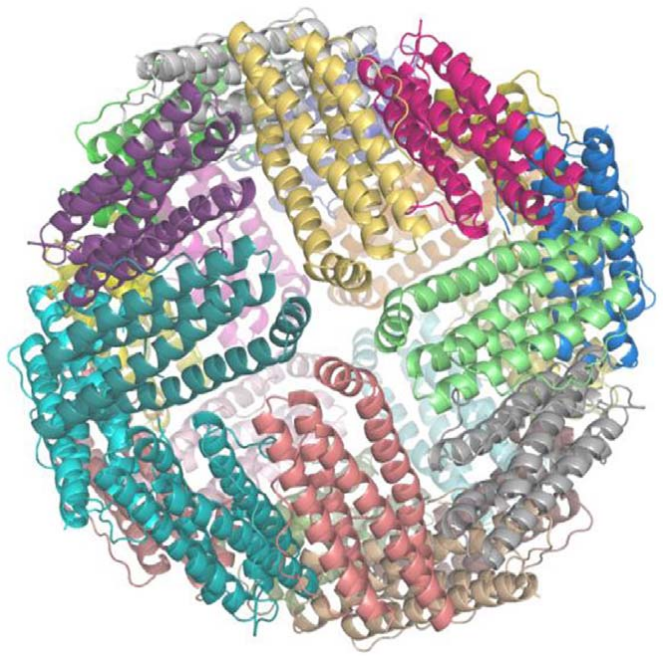
Crystal structure of an asymmetric unit (24 subunits) of Mtb BfrB-WT. The ribbon diagram of the contents of asymmetric unit (24 subunits) of Mtb BfrB-WT crystal down the 4-fold channel corresponds to the near spherical biological form of the protein.

**Figure 3:**
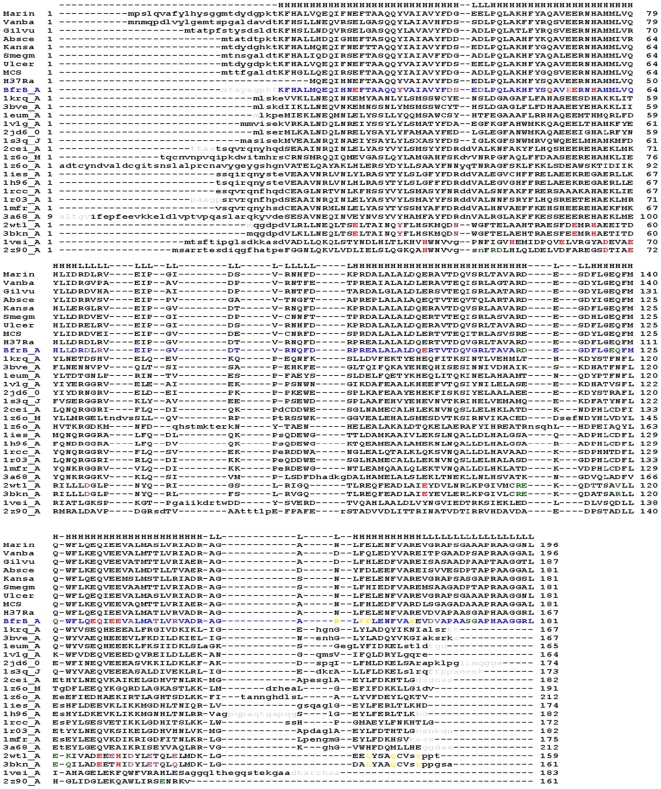
Multiple sequence alignment of representative ferritins. Annotated ferritin sequences from other mycobacterial species are placed above the Mtb BfrB sequence (blue) whereas the sequences of ferritin representatives with known structures (defined by their PDB code and chain ID) are placed below. The first and the last residue numbers are indicated before and after each sequence. Uppercase means structurally equivalent positions with Mtb BfrB. Secondary structure assignment for Mtb BfrB_A is shown at the top of the sequence (H = helix, L = coil). The lower case residues in grey color do not have coordinates in their respective PDB files. They are shown here for completion of the sequence. Amino acids in mycobacterial ferritins involved in ferroxidase activity, at the 4-fold, 3-fold and B-pore are highlighted in red, yellow, green and plum colors respectively.

**Figure 4:**
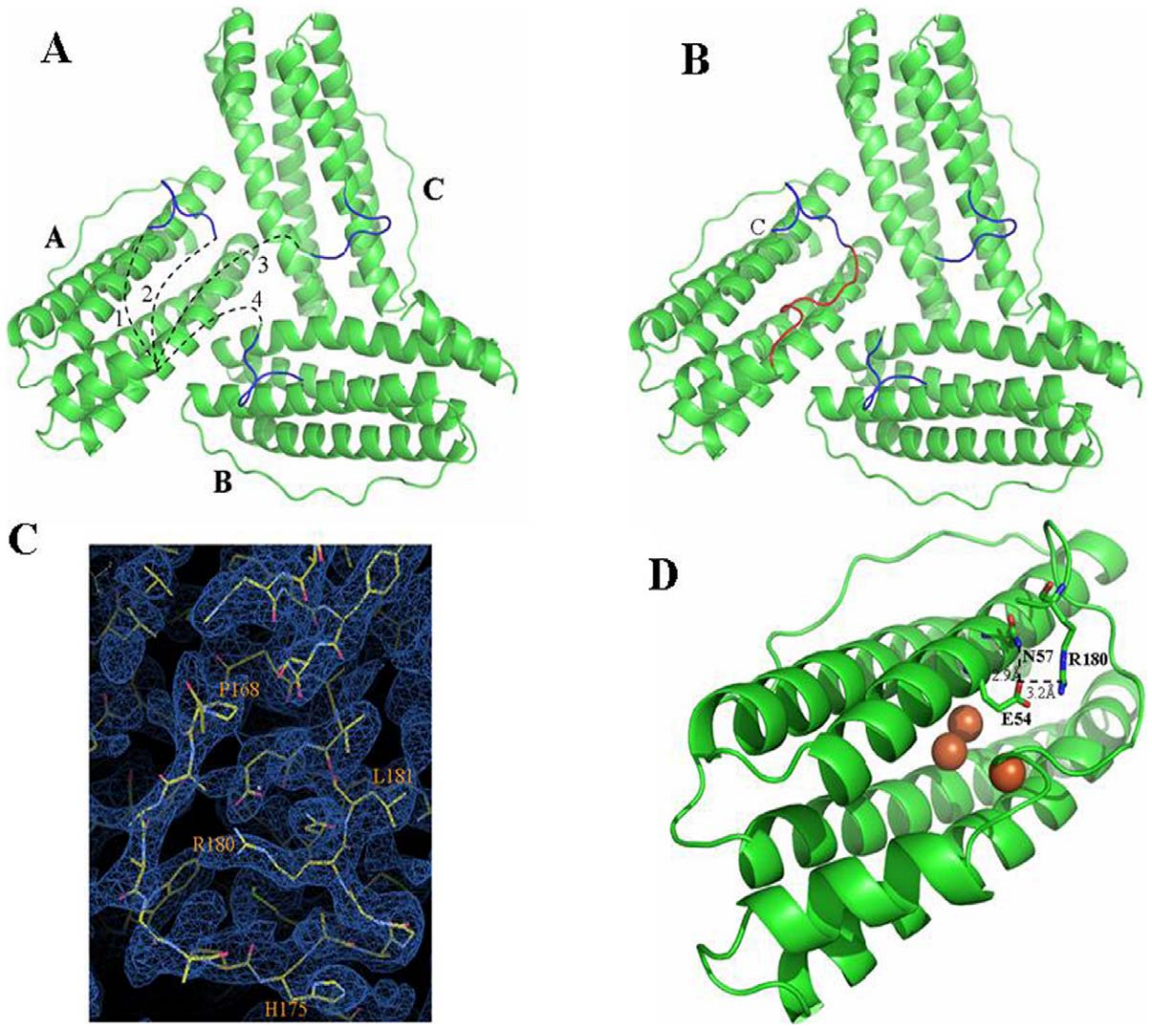
C-terminus Mtb BfrB. (A) Ribbon representation of the 3-fold channel, composed of subunits A, B and C when viewed from inside of the molecule. Unambiguous region of the subunit (10–163) is shown in green whereas poly-ala stretch for residues 174–181 (C_rigid_) is shown in blue. Due to absence of any electron density for residues 164–173 (C_flexible_), initial uncertainty existed in threading this far C-terminus end with the rest of the subunit. Many possibilities existed that are numbered 1 to 4 in figure 4A (drawn by black dashed lines). For example: subunit A could be connected to the same C_rigid_ either as shown in model 1 or as shown in model 2. Equal probability existed for inter-subunit connectivity as subunit A could be linked with C_rigid_ stretch located at subunit C (model 3) or with C_rigid_ located at subunit B (model 4). (B) Final Mtb BfrB model built as per the model possibility 2 from figure 4A. Ribbon structure corresponding to C_flexible_ residues 164–173 in subunit A of Mtb BfrB as viewed from inside of the molecule is shown in red color and the C-terminus of the monomer is labeled. (C) 2Fo-Fc electron density map for C_flexible_ 164–173 region of subunit A contoured at 1.0σ show side chain density for residues P168, H175, R180 and L181 and provide confidence in the orientation and connectivity. (D) View of Mtb BfrB monomer in cartoon representation showing interaction of C-terminus with B-helix. The interactions of E54 with N57 and R180 are shown with black dashed line. The iron atoms (shown as brown spheres) are taken from *Pyrococcus furiosus* structure (2jd6:0) and represent the putative bound iron at the ferroxidase centre.

The model constituting of residues 10–163 (in all 24 subunits) was built without any ambiguity (Figure 2) but the C-terminal stretch (164–181) posed a problem. This stretch is extended as compared to most of the other members of the family and is highly specific to mycobacteriaceae (Figure 3). Definite electron density could be traced for the far end of the C-terminus (174–181) in all subunits and hence poly ala for this stretch was built in. This stretch lies on a 3-fold junction of BfrB (Figure 4A). Due to the discontinuity in the protein backbone, it became difficult to decide the orientation of this stretch, as no significant difference was observed in R_free_ on building it either way. Moreover, uncertainty existed in assigning this stretch to a particular subunit, since many possibilities (intra- as well as inter-subunit) existed as depicted with black dashed lines in Figure 4A. Structure of another lower resolution dataset (3.8 Å) of BfrB tagged with a Strep-Tactin tag at the C-terminus exhibited reasonable contiguous density for residues 164–173 in some subunits (data not shown) and provided evidence for alternative 2 (as defined in Figure 4A). This structure was modeled in the higher resolution wild type BfrB structure but unfortunately exhibited density in only one subunit. As the C-terminus 174–181 stretch appears robust in all 24 subunits, it is being labeled as C_rigid_ (blue in the Figure 4B). However, the minimal density for the C-terminus 164–173 in other subunits indicates that this region of the C-terminus is flexible and hence is being labeled C_flexible_ (red in Figure 4B). The electron density for the residues (H175, R180 and L181) belonging to C_rigid_ (Figure 4C) provides confidence that the model shown in Figure 4B has a good chance of being in register. Interaction of R180 with E54 and N57 could be the reason for C_rigid_ adopting this stable structure (Figure 4D). Interestingly, this portion of the C-terminus that loops back to interact with B-helix at the 3-fold junction is positioned at the bottom of the ferroxidase centre.

### Structure of Mtb BfrB: Conservation and variation

Similar to the assembly of other ferritins from eukaryotes and prokaryotes, the macromolecular Mtb BfrB exhibits a cage-like hollow shell constituting of 24 monomers that are related by 432 symmetry (Figure 2). In the final structure, all subunits exclude the N-terminal 1–9 residues. Further, with the exception of subunit A, C_flexible_ is not modeled in rest of the subunits. A structure based multiple sequence alignment of Mtb BfrB with homologous ferritins from all kingdoms shows low sequence identity ranging from 11 to 28% (Table 2, Figure 3), yet a very high degree of structural similarity. The solvation free energy of a protein molecule reflects the effect of solvent (water) associated with its structure and is often correlated with its thermal stability [42], [43]. The solvation energies computed for single subunits of listed structures (Table 2) reveal that among the mycobacterial ferritins, Mtb BfrA appears to be more stable, an observation that needs further investigation.

**Table 2:** A comparative analysis of subunit structure of Mtb BfrB with representative ferritins from archaea, eubacteria and eukarya using the DaliLite -pairwise option vs 3.1.

Organism (ferritin annotation)	PDB-Chain	Z score	RMSD (Å)	N_aligned_	Total N	Identity (%)	Solvation Energy (kcal/mol)	Reference
**Eubacteria ferritins**								
*M. tuberculosis*	BfrB_A	-	-	-	181		−3956.8	This study
*C. jejuni*	1krq_A	22.9	1.1	156	164	24	−3651.4	Unpublished
*H. pylori*	3bve_A	22.4	1.2	157	173	19	−3343.4	[18]
*E. coli*	1eum_A	22.4	1.3	154	161	19	−4140.6	[15]
**Archaebacteria ferritins**								
*P. furiosus*	2jd6_O	22.4	1.8	156	167	27	−3855.4	[16]
*A. fulgidus*	1s3q_J	22.1	1.6	157	164	27	−4576.6	[17]
*T. maritima*	1vlg_A	22.0	1.7	157	164	24	−4473.6	Unpublished
**Eukaryotic ferritins**								
Human H -chain	2cei_A	20.8	1.2	151	172	25	−3798.2	[7]
Insect H-chain	1z6o_M	19.1	1.5	151	191	27	−3528.0	[14]
Insect L-chain	1z6o_A	17.7	1.7	150	212	17	−3642.5	[14]
Horse spleen	1ies_A	20.6	1.2	152	174	18	−2991.4	[11]
Mouse L-chain	1h96_A	20.5	1.2	151	166	19	−3062.5	[74]
Bullfrog L ferritin	1rcc_A	20.0	1.8	155	171	23	−2435.0	[9]
Human Mitochondria	1r03_A	20.7	1.2	151	170	25	−4213.3	[75]
Bullfrog M ferritin	1mfr_A	20.3	1.6	154	171	25	−3999.9	[8]
Soybean	3a68_A	20.2	1.2	153	194	28	−4673.3	[13]
**Mycobacterial heme-containing bacterioferritins (Bfrs)**								
*M. tuberculosis* (BfrA)	2wtl_A	17.0	2.3	148	161	20	−5109.5	[38]
*M. smegmatis* (Bfr)	3bkn_A	17.2	2.2	148	161	20	−4388.7	[76]
**Mycobacterial DNA binding proteins from starved cells (Dps)**								
*M. smegmatis* (Dps1)	1vei_A	13.0	2.4	131	175	11	−4189.0	[58]
*M. smegmatis* (Dps2)	2z90_A	12.7	2.4	130	161	12	−3964.8	[77]

**Figure 5:**
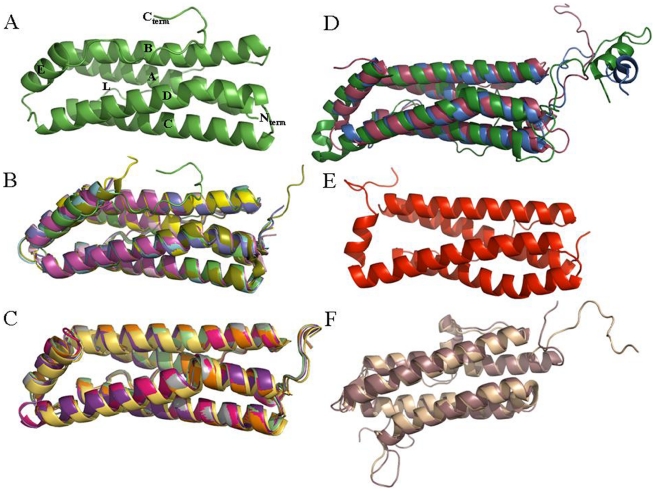
Ribbon diagram of the subunit structure of ferritin family representatives. The PDB code followed by the Chain ID and the color of the representative is indicated in parenthesis. (A) Mtb BfrB subunit (in green) where all the five helices (A–E), L-loop and the two termini are labeled. (B) Mtb BfrB subunit superimposed with ferritins from archaea and eubacteria - *Campylobacter Jejuni* (1krq:A; cyan), *Escherichia coli* (1eum:A; magenta), *Pyrococcus furiosus* (2jd6:0; yellow), *Archaeoglobus fulgidus* (1s3q:J; pink), *Helicobacter pylori* (3bve:A; olive green), *Thermotoga maritime* (1vlg:A; purple blue). (C) Superpimposed eukaryotic mammalian ferritins from human H ferritin (2cei:A; orange), human mitochondrial ferritin (1r03:A; pale green), horse spleen L ferritin (1ies:A; hot pink), mouse L ferritin (1h96:A; mustard). (D) superimposed eukaryotic non-mammalian ferritins from soybean (3a68:A; blue) and secreted insect (*Trichoplusia ni*) ferritin with unique N-terminal extensions in the H (1z6o:M; raspberry) and L subunits (1z6o:A; forest green). (E) Mtb BfrA subunit (1wtl:A; red). (F) Superimposed Dps1 (1vei:A; brown) and Dps2 (2z90:A; wheat) from *Mycobacterium smegmatis*.

Despite low sequence homology across kingdoms, the ferritin structural fold is highly conserved. Figure 5 presents cartoon diagrams of the subunit structures of Mtb BfrB, archaeal and eubacterial ferritins, mammalian and non-mammalian eukaryotic ferritins as well as mycobacterial heme-containing Bfr and Dps proteins, illustrating the striking similarity in their global subunit folds.

The results of a structure based multiple sequence alignment with the program DALI [44] shows the highest Z-score (∼22.0) for Mtb BfrB with ferritins belonging to eubacteria and archaea.

The conserved region of each BfrB subunit is composed of the A-helix (H12 to S38), B-helix (P42 to D68), BC loop (R69 to R87), C-helix (P88 to D115), D-helix (F119 to R148) and E-helix (L153 to E163). The RMSD's in backbone positions of aligned residues when BfrB is superposed (in pairs) with known ferritin structures vary between 1.1 Å and 1.8 Å, whereas, when superposed with mycobacterial Bfr and Dps members, the value is slightly higher and corresponds to 2.2–2.4 Å (Table 2). This variability is reasonable as the latter two are paralogues from two distinct ferritin subfamilies. The consistent structural features among all three types of mycobacterial ferritins are helices (A–D) forming the characteristic four-helical bundle. The placement of the short E helix varies within the subfamilies. In Mtb BfrB and other ferritins, this helix lies 60° to the central axis of the four-helical bundle, whereas, in Mtb and *M. smegmatis* heme-containing Bfrs and Dps proteins, this helix is roughly perpendicular to the axis (Figure 5E). In all ferritins and Bfrs, this short helix is located at the C terminus and defines the 4-fold axis in the oligomeric assembly, whereas in Dps proteins, the short helix is located between the B and C helices and is positioned at the 2-fold symmetry axis. As evident from figures 3 and 5, the N and C terminii along with BC and DE loops are the most variable segments both in terms of length and composition within the ferritin family.

**Figure 6:**
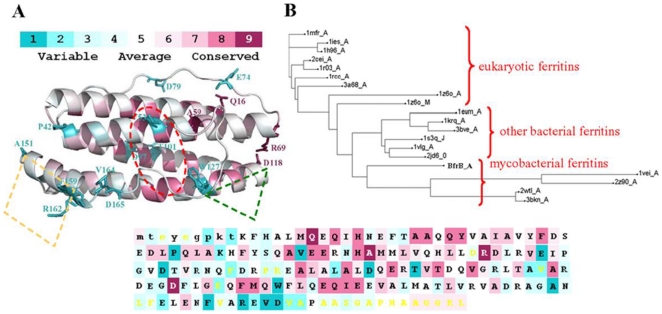
A ConSurf analysis for Mtb BfrB structure. The residues in the 3D cartoon structure as well as in the primary sequence are colored by their conservation grades using the nine grade color-coding bar, with turquoise-through-maroon indicating variable-through-conserved. Yellow colored residues in the sequence panel denote regions that were assigned conservation level with low confidence due to insufficient data and hence have been excluded from the analysis. Highly conserved and variable residues are marked and depicted as sticks. Location of the conserved ferroxidase site as well as the variable 4-fold and 3-fold channels is marked with dotted oval (red), diamond (yellow) and triangle (green) respectively. The analysis was carried out using one subunit of Mtb BfrB structure and multiple sequence alignment given in figure 3. (B) A phylogenetic tree of ferritin representatives constructed by the ConSurf server.

The structural and functional importance of a residue in a structure will govern its evolutionary conservation. Hence, conservation analysis of amino acid positions among the ferritin family members with known structures (listed in Figure 3) was performed with the program Consurf [45]. Conservation score, grouped into a nine-color grade scale (where the most conserved and variable positions are marked in maroon and turquoise, respectively), is mapped onto the 3D structure of Mtb BfrB structure (Figure 6A). The figure reveals that the most conserved positions are Q16, A59, R69 and D118; with P42, V53, E74, D79, D97, T101, W127, A151, F159, R162, V164, and D165 being the most variable positions.

The importance of these positions for the protein's structure or function will be discussed in the later section. Residues Y29, E54, E55, H58, E99, E131, Q132 and E134 representing the ferroxidase site exhibit conservation grade of 8, while residues E22 and Q51 also involved in metal binding display conservation grade of 6. E135 is the only variable catalytic residue with a low conservation score of 4. Further, a phylogenetic tree based on multiple sequence alignment (Figure 3) is reconstructed by using the neighbor-joining algorithm (Figure 6B).

### The ferroxidase centre

**Figure 7:**
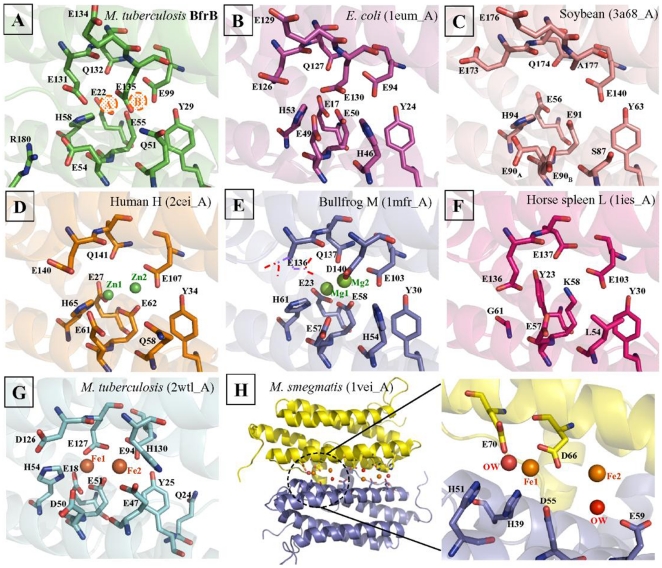
Metal coordinating residues at the ferroxidase center of ferritin representatives from various organisms. (A–H) The source of ferritin is indicated on the right top corner of each figure along with PDB ID and chain ID in parenthesis. The residues involved in the ferroxidase center are mentioned in each figure. The metal atoms are shown as spheres in their atom colors wherever their coordinates are available in the PDB files. The presumed bound irons at A- and B- site in Mtb BfrB are shown with dotted brown spheres.

The initial step in the process of iron uptake by ferritin involves the oxidation of ferrous iron at the di-iron centre composed of metal-binding sites A and B, liganded mostly by acidic residues [46], [47]. The iron occupancy of site A is found to be higher than that of site B [6]. Some ferritins include a third iron-binding site called the “C-site” also formed mostly by Glu residues [15], [48]. The environment of representative ferroxidase centres of Mtb BfrB, *E. coli* Ferritin A, soybean ferritin, human H-chain ferritin, bullfrog M ferritin, horse spleen L-chain ferritin, Mtb BfrA and *M. smegmatis* Dps are shown in Figure 7. The residues involved in metal binding at the di-iron site are widely conserved across the ferritin family with structurally equivalent placements as evident from figures 3, 6A and 7. Subtle differences such as conformational or residue replacement is apparent within the classical ferritins. Also as recognized, the location of di-iron site varies between ferritin/Bfr and Dps subfamilies. In the former, the centre is located in a four-helix bundle core whereas in the latter, it is located at the interface between two subunits related by 2-fold symmetry. In general, Glu, Tyr and His residues that are mostly conserved in all the ferritins stabilize the two iron ions at the di-iron centre (Figure 3). In the present crystal structure of Mtb BfrB (Figure 7A) the metal site is not occupied, but the overall structure of the site is nearly identical to that of all the illustrated homologues. The residues involved in the ferroxidase centre correspond to the E22, E55 and H58 as ligands for the A-site and E99, E135 (along with E55) as ligands for the B-site. In *E.coli* ferritin, the well-confirmed third C-site comprises residues E49, E126, E129 and E130 (Figure 7B). In Mtb BfrB, these residues correspond to E54, E131, E134 and E135, respectively. E54 in Mtb BfrB has a flipped orientation that is stabilized by formation of a salt bridge with R180 (unique to mycobacterium species, Figure 3) suggesting an altered C-site in Mtb BfrB.

### Electrostatic factors

**Figure 8:**
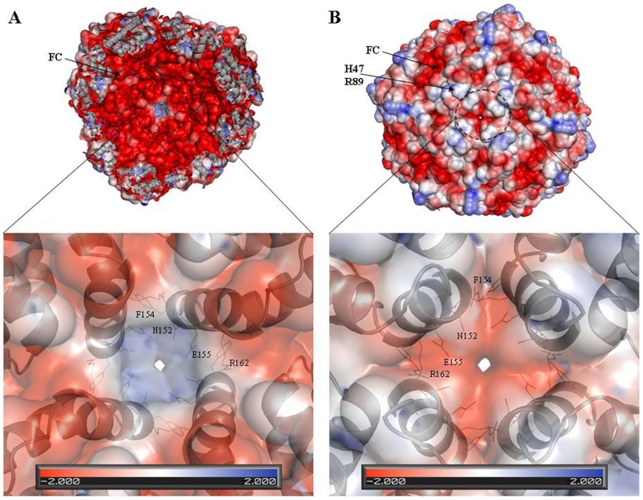
Molecular surface along the 4-fold channel of Mtb BfrB. The surface is colored according to the values of electrostatic potential positioned as viewed from the (A) interior and (B) exterior of the octahedral shell. The molecular surface of ferritin was cut in half by an imaginary plane for the interior view. The insets show the close up view and the conformation of the residues at the 4-fold junction.

**Figure 9:**
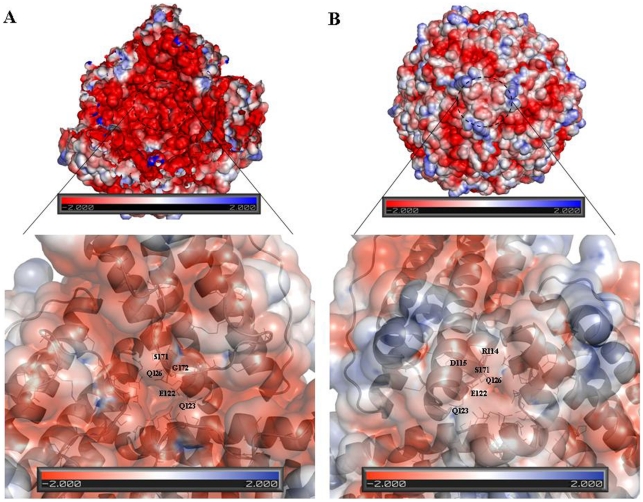
Molecular surface along the 3-fold channel of Mtb BfrB. The surface is colored according to the values of electrostatic potential positioned as viewed from the (A) interior and (B) exterior of the protein. The molecular surface of ferritin was cut in half by an imaginary plane for the interior view. The insets show the close up view and the conformation of the residues at the 3-fold junction.

Electrostatic gradients are important for the overall functioning of the protein [49]. Electrostatic models using the Mtb BfrB structure exhibit predominantly negative potentials lining the interior surface of the octahedral shell (Figure 8A). Yet surprisingly, the mouth of the 4-fold channel (opening into the hollow cavity) shows positive charge, in stark contrast to the rest of the surface. This channel (Fig. 8A, inset) comprises of residues N152, F154, E155 and R162. All these residues indicate variable conservation grades ranging from 1 to 4 as per the ConSurf analysis (Figure 6A). The exterior surface of the ferritin molecule as viewed down the 4-fold axis shows well defined regions of both positive as well as negative potential (Figure 8B). Interestingly, from outside, the entrance to the 4-fold channel displays negative values of the potential. The other major region displaying negative potential is the expected ferroxidase centre. The region of positive potential, adjacent to the negative ferroxidase site can be attributed to R89 and H47, again variable spots with conservation grade of 3 and 5, respectively (Figure 6A). Similarly, looking down the 3-fold axis, the interior surface flaunts negative electrostatic values whereas exterior surface illustrates both positive and negative values (Figure 9).

**Figure 10:**
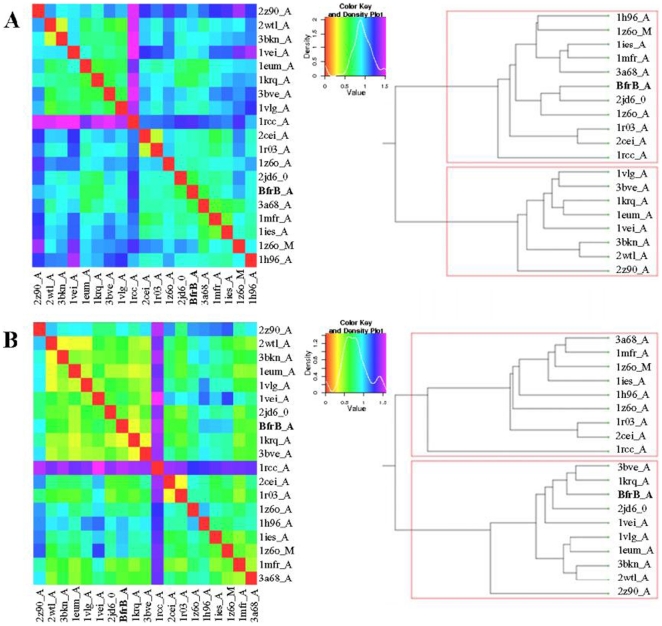
Comparison of the electrostatic potentials of Mtb BfrB with homologous structures used in **figure 3**. webPIPSA is used to compare the electrostatic potentials of (A) single subunit and (B) ferroxidase centre of specified ferritins. It was also used to calculate a distance matrix according to the all pairwise distances between their electrostatic potentials. The distance matrix is displayed using a color code from red (small distance) to blue (large distance). In addition, ferritins from different species are clustered according to the relations between their electrostatic potentials and the clustering is displayed in a tree-like dendogram along the side of the distance matrix.

Figure 10 displays the results of PIPSA (Protein Interaction Property Similarity Analysis) investigation, carried out for calculating the electrostatic potentials for Mtb BfrB and all other homologous ferritin structures employed in Figure 3. The result of pairwise comparison of the electrostatic potentials of a single subunit of all the ferritin family affiliates is displayed as a colorized matrix in Figure 10A. Red/orange colors indicate similar potentials, whereas blue color point towards proteins with more distant electrostatic potentials. In addition, a tree-like dendogram along the side of the image clusters the proteins into groups of similar electrostatic potentials and displays the relation between them. Here only the relatedness of the leaf nodes is displayed without making assumptions about evolutionary timeline. The length of the tree branches in the dendogram is not a quantitative measure of relatedness but is scaled for optimal display. The figure shows that nearest neighbours of Mtb BfrB are the homologues from *Pyrococcus furiosus* (2jd6_0) and soybean (3A68_A) while the farthest is that of L-chain ferritin from red cells of Bullfrog (1rcc_A). Distances range from 0.37417 to 1.54013. Similar comparison restricted to the ferroxidase centres of ferritins in Figure 10B indicates that ferroxidase centre of BfrB most highly resembles those of *Campylobacter jejuni* (1krq_A) and *Helicobacter pylori* (3bve_A) while the largest difference again lies with that of Bullfrog L-chain ferritin (1rcc_A). Distances range from 0.32249 to 1.60437.

### 4-fold channels, 3-fold channels and the B-pore

**Figure 11:**
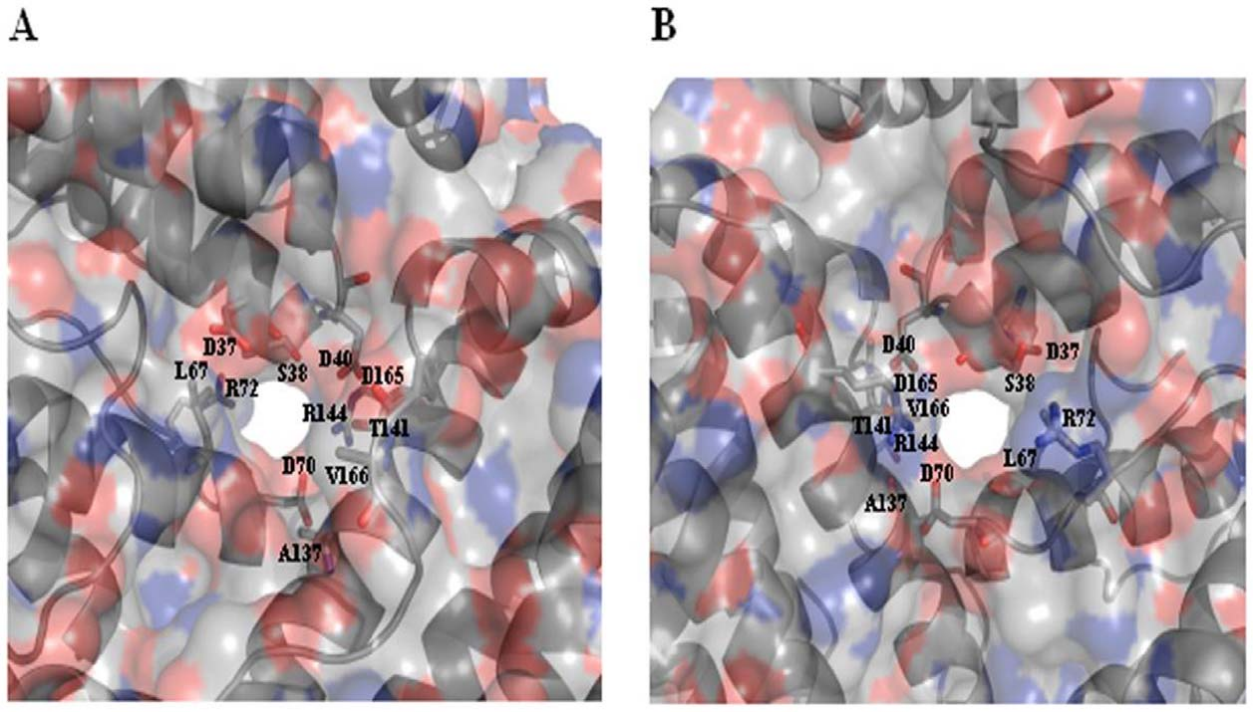
Molecular surface along the B-pore of Mtb BfrB. The molecular surface representation of the B-pore as viewed from the (A) interior and (B) exterior of the protein. The surface is colored by atom type.

The exterior and interior of the ferritin shell are primarily connected via channels along the 4-fold and 3-fold symmetry axes. These channels are known to be involved in the entry and exit of iron ions [50], [51], [52]. Despite a very similar overall topology, a remarkable heterogeneity is observed for the subunits of different classes of the ferritin superfamily, indicative of different potential functions executed by its various members [5]. As illustrated in Figure 8, the 4-fold channel of Mtb BfrB is lined with N152, F154, E155 and R162, where the exterior mouth of the channel is negatively charged while the interior mouth is positively charged. The variability in the composition of 4-fold channels among the ferritin family members is obvious from Figure 3 (residues highlighted in yellow color) and Figure 6A. The 3-fold channel is lined with R114, D115, E122, Q123, Q126 along with carbonyl oxygens of S171 and G172 pointing into the channel (Figure 9). Positions other than Q123 and Q126 (having conservation grades of 6 and 7, respectively) display variability amongst the ferritin members (Figure 3 and Figure 6A). S171 and G172 residues form part of the extended C-terminal region. Negative and positive charges may create an electrostatic field to direct ferrous ions into the interior of the protein shell similar to that proposed for recombinant human H-chain ferritin [49]. A pore large enough to accommodate an iron atom is created from residues (highlighted in plum color in Figure 3) from three subunits namely, D37, S38, D40 from one subunit, L67, D70, R72 from the second and A137, T141, R144, D165 and V166 from the third subunit (Figure 11). With the exception of T141, D165 and V166 that present variability, most of these residues show moderate level of conservation. This pore, lined mostly with negatively charged residues N34, D66, D132, E135, T136 and E139, also exists in Mtb BfrA [38] and appears to be the most likely route for iron entry/exit to/from the Bfr cavity.

### Intra- and inter-subunit Interactions

The extensive and cooperative intra- and inter-subunit interactions are responsible for folding/stability of ferritin assembly as illustrated by a study analyzing the stability difference between human H- and L-chain ferritins [53]. To define more specifically the stabilizing interactions in the structure of Mtb BfrB, all the energetically significant interactions are compiled in Table 3 and analyzed with respect to conservation with other representative structures. The salt bridge between R69 of the BC loop and D118 of the short CD loop is not only a conserved interaction in ferritins but is also present in mycobacterial Bfrs and Dps proteins, although in the other subfamilies the Arg is not from the structurally equivalent position of R69. These residues are in proximity to the 3-fold junction of folded subunits and form a “gate” that responds to changes in the environment by “opening” or “closing” a channel pore so as to increase or decrease the rates of ion transport. The most fascinating interaction entailing further discussion involves residues F154 surrounded by E155, R148 and R162 resulting in anion-pi-cation synergy. The interactions that appear specific to mycobacterium species involve D40, R114, D115, W127, R144, V145, F154, E155, F159, R162 and the extended C-terminus.

**Table 3:** Intra- and inter-subunit interactions in Mtb BfrB structure and relative conservation among representative ferritins.

Intra-subunit interactions					
Residue pair	Distance (Å)*	Conservation	Residue pair	Distance (Å)	Conservation
Salt Bridges			Pi-Cation		
K10 : E116	3.9	++, C^Myco^	F85 : R82	5.7	+
E54 : R180	3.7	+	F159 : R148	6.5	++, C^Myco^
R69 : D118	3.2	+++	Pi-Sigma		
R82 : E90	3.5	+	H12 : L71	3.9	C^Myco^
R89 : D147	3.1	+, C^Myco^	W127 : A173	3.5	C^Myco^
R114 : D115	3.5	+, C^Myco^	W127 : P174	3.6	C^Myco^
R162 : E163	2.6	+, C^Myco^	F159 : V145	3.9	C^Myco^
Pi-Pi					
H65 : F124	4.0	++, C^Myco^			

Inter-subunit interactions					
Salt Bridges			H bonds		
E39 : R148	2.7	+	Q27 : P76	3.1	+, C^Myco^
D40 : R144	3.4	C^Myco^	D40 : T141	2.8	+, C^Myco^
D40 : R148	2.7	C^Myco^	Q43 : E163	3.1	+, C^Myco^
R144 : D70	3.4	+	I75 : N83	3.1	+, C^Myco^
R162 : E157	3.9	C^Myco^	V78 : V78	2.7	+++
Pi-Cation			Q123 : Q130	2.5	+
F154 : R148	4.0	C^Myco^	N152 : E155	3.2	+
F154 : R162	6.0	C^Myco^	R162 : N158	2.9	+
F119 : R114	5.0	C^Myco^	Pi-Anion		
Pi-Pi			F154 : E155	3.2	+
F154 : F159	4.9	C^Myco^			

+/++/+++: Variable/Average/High conservation; C^Myco^: conserved in mycobacterial ferritins;

*: The listed distances are as observed in (intra- or inter-) subunit A of Mtb BfrB.

### Influence of the extended C-terminus on the stability of Mtb BfrB

**Figure 12:**
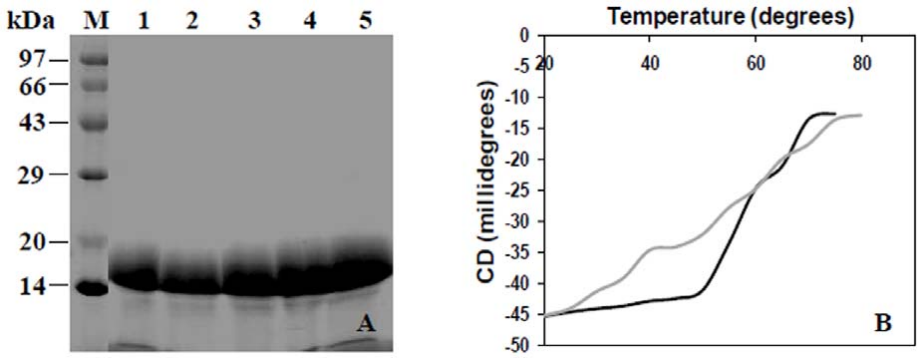
Purification and Thermal denaturation of Truncated BfrB. (A) Analysis of the purified truncated BfrB on a 12.5% SDS-polyacrylamide gel. M- Molecular weight markers, lanes 1–5 – purified protein. (B) Thermal denaturation curve of full length and truncated BfrB carried out by using circular dichroism. Black line denotes the full length BfrB while grey line represents truncated BfrB.

To dissect the role of the extended C-terminus region in the Mtb BfrB, recombinant truncated BfrB was generated. The truncated protein (having 1–167 amino acids, apparent molecular weight ∼18 kDa) was purified similarly as described for the full length protein (Figure 12A). It was observed that removal of the extended C-terminus had no effect on the formation of macromolecular assembly and overall structure of the protein as analyzed by the gel filtration chromatography, native gel analysis and circular dichroism spectra (data not shown). However, the thermal denaturation studies revealed that the presence of extended C-terminal end provides stability to the protein, which is in agreement with the crystallographic analysis. Truncated BfrB starts unfolding on exposure to even a very low temperature of 30°C whereas the native protein remains almost unaffected till 50°C before denaturing rapidly (Figure 12B).

### Influence of the extended C-terminal region of BfrB on iron oxidation and release

**Figure 13:**
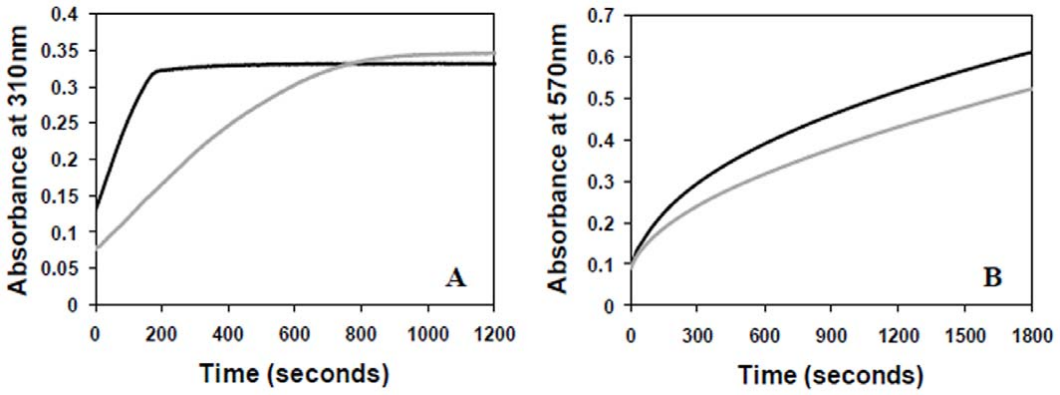
Influence of the extended C-terminal region of BfrB on iron oxidation and release. (A) Iron oxidation progress curve of full length and truncated BfrB where the oxidation of Fe(II) to Fe(III) was monitored by an increase in the absorbance at 310 nm. (B) Comparison of the release of mineralized iron from full length and truncated BfrB where the released iron was measured as Fe(II)-Ferrozine complex at 570 nm. Black line denotes the full length BfrB while grey line represents truncated BfrB.

Next, we monitored iron oxidation by measuring the increase in the absorbance at 310 nm resulting from the formation of Fe(III) species in a reaction containing 1∶500 ratio of protein∶iron. Comparison of the native BfrB with the truncated protein showed that removal of the extended C-terminus causes a 3.5-fold reduction in the oxidation rate of Fe(II). While the native protein oxidized all the iron within the first 3 minutes, the truncated protein required 13 minutes for the same (Figure 13A). Release of mineralized iron from ferritin was measured by the reduction of Fe(III) to Fe(II) in the presence of reductant sodium ascorbate followed by complexing of the released Fe(II) with ferrozine reagent resulting in a pink coloured complex, which could be measured spectrophotometrically at 570 nm. The truncated protein exhibited a 20% reduction in the release rate as compared to the native protein (Figure 13B).

## Discussion

The Mtb ferritin BfrB adopts the highly conserved structural architecture of the ferritin family, consisting of 24 subunits arranged in an octahedral symmetry enclosing a hollow cavity for the storage of iron. Multiple sequence alignment revealed the presence of an extended C-terminal region comprising of 18 residues in mycobacterial ferritins. The structure of Mtb BfrB showed the presence of this extended C-terminal region. Although uncertainty existed in the side chain conformation of the C_flexible_ region (the proximal region of the stretch), the electron density for the C_rigid_ (the distal region) left no ambiguity in the structural definition of this expanse (Figure 4C). Moreover, thermal denaturation curves confirm that the presence of C-terminus renders stability to Mtb BfrB (Figure 12B). This is also in agreement with the presence of C_rigid_ stretch (174–181) with residue R180 interacting with E54 and N57, as observed in the crystallographic analysis, thus this stretch (174–181) seems to confer additional rigidity to the protein.

**Figure 14:**
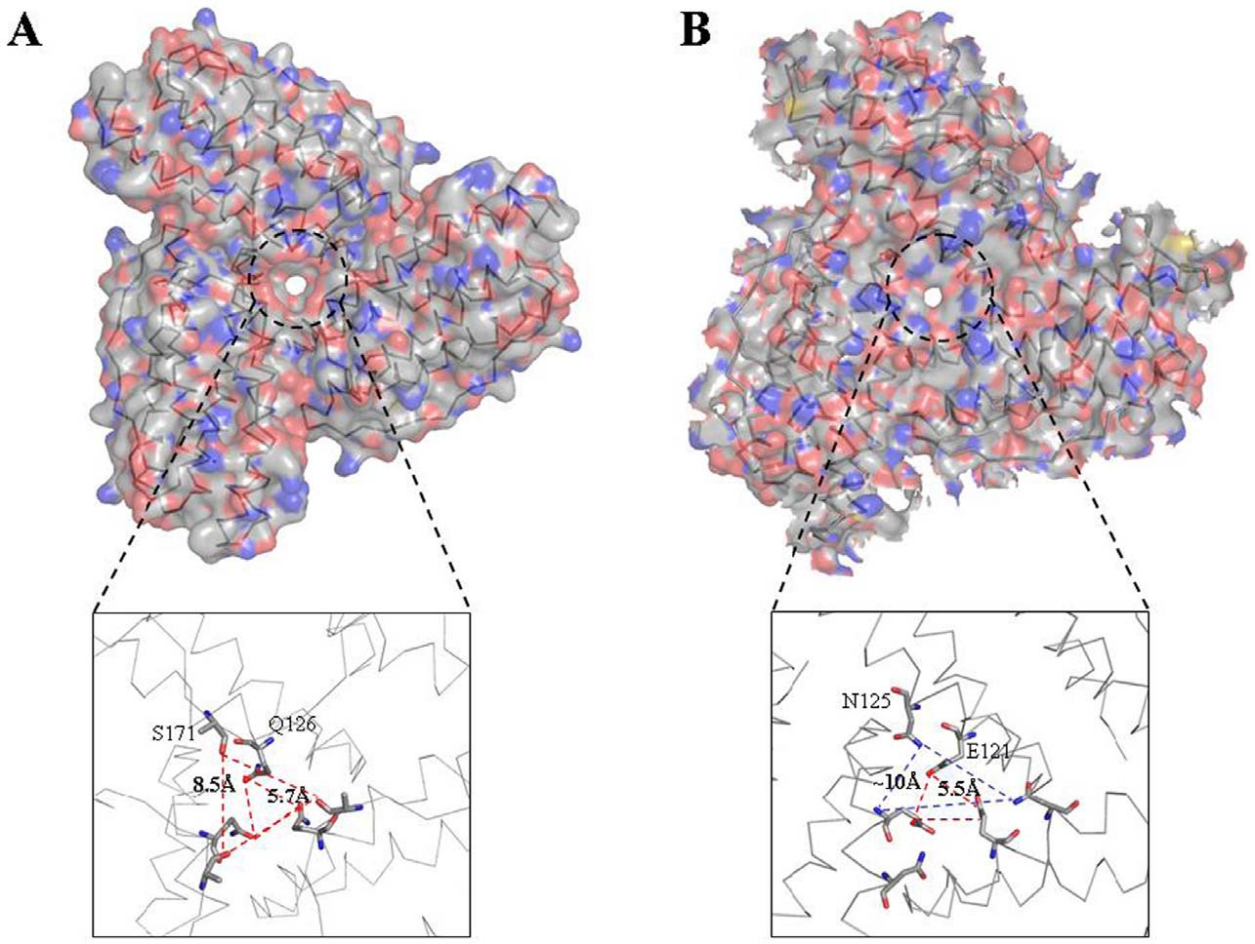
Comparison of trimer junction of *M. tuberculosis* and *P. furiosus* ferritins. (A) The surface representation of 3 subunits of BfrB at the 3-fold junction when viewed from inside of the molecule to illustrate the effect of its extended C-terminus. The arrangement of residues, Q126 and S171 (stick representation) involved in formation of the channel mouth are shown in the inset. (B) Surface representation of similar region in *Pyrococcus furiosus* where the pore is constituted by E121 (towards the ferroxidase centre) and N125 (towards inner core).

Although the ferritin sequence from *Pyrococcus furiosus* also has the unusually extended C-terminus, the complete structure for this extended stretch is not available (Figure 3). Analysis of the funnel-shaped 3-fold channel divulges some interesting insights (Figure 14). This junction of three ferritin subunits is known to be a dynamic aperture that regulates iron entry/exit *in vivo*. The lining of the three fold channel in the case of *Pyrococcus furiosus* is marked by the presence of positively charged residues, whereas the three-fold lining in case of Mtb BfrB is dominated by the presence of negatively charged residues (Figure 14) suggesting that the predominance of these negative charges would enhance iron binding capacity of the latter and hence, would lead to a higher inflow of iron towards the ferroxidase centre thus resulting in more efficient ferroxidase activity. In addition, the presence of extended C-terminus residue S171 at the three-fold channel in Mtb BfrB enhances its negative character and may further increase the efficiency of the ferroxidase centre. In fact our studies on the comparison of ferroxidase activity exhibited by Mtb BfrB and its C-terminus truncated version show that this indeed is the case. A drastic 3.5 fold reduction in the oxidation rate exhibited by the truncated Mtb BfrB in comparison to the native protein along with the crystallographic observations stated above imply the role of this C-terminal region in facilitating the iron entry from the three-fold channel towards the ferroxidase centre. In addition, a ∼20% reduction in the rate of iron release observed in the absence of C-terminus reflects the association of this region with the exit of stored iron from protein's cavity as well; however, more detailed investigations would be required for a better understanding of these phenomena. A study by Theil and coworkers proved that mutation of conserved Leu (residue 134) to Pro increased disorder at the trimer subunit junction and resulted in enhanced iron exit [54]. In Mtb BfrB, the extended C-terminus has two prolines (^164^VDVAPAASGAP^174^) and could be playing the analogous role in regulating iron exit. Possibly, depending on the cellular factors, this loop is akin to a camera shutter, regulating iron oxidation/reduction or entry/exit rate. Mtb BfrB is known to get highly induced under high iron stress conditions and it seems advantageous for the cell to have a highly efficient ferroxidase activity to quickly respond to this stress and quench the iron efficiently. The biological importance of a residue often correlates with its level of evolutionary conservation within the protein family [45]. The multiple sequence alignment of 20 representative ferritin structures, as presented in Figure 3, was used for the ConSurf analysis to determine highly conserved residues that are potentially necessary for either structural integrity or biological activity. The computed degree of conservation for each residue was then mapped onto the structure of Mtb BfrB (Figure 6). The conserved patches essentially fall in the centre of the four-helix bundle indicating conservation of residues at the ferroxidase site. The most highly conserved residues are Q16, A59, R69 and D118, out of which the side chain of Q16 make hydrogen bonds with main chain atoms of residue E74 (located in the BC loop) and hence appears to be important for stabilizing and positioning of a loop that is essential for maintenance of the dimer interface. The side chain of R69 forms an intra-subunit salt bridge (3.27 Å) with D118 and is a conserved ion-pair interaction in the ferritin family. Structurally equivalent R72 and D122 in horse spleen L-chain ferritin have been recently identified as gated protein pores that control the transfer of ferrous iron into and out of the ferritin nanocage. Disrupting the ion pair between D122 and R72 changed pore function considerably [55]. Both the length and the sequence in the CD loop have been noted as important for pore function. As this loop is pointing into the 3-fold funnel with D122 at the base, it is possible that this residue blocks reduction of the ferric mineral when the ferritin gated pore is folded or closed. These ferritin gated pores are recognized as structurally and functionally homologous to gated pores in ion channel proteins embedded in cell membranes. High evolutionary conservation of the pore gates argues for the presence of physiological ligands/regulators for these gates that hold them either closed or open, depending on the biological iron need *in vivo*. The same study [55] also identified another pore gate involving the L110 and L134 residue pair. In Mtb BfrB, structurally equivalent residues V106 and L129 may perform the analogous function of pore gate. However, it is imperative to point out here that C_flexible_ stretch lies between the V106/L129 residue pair and the inner mineral cavity, opposing the requirement of this residue pair to function as a pore gate. Moreover, L129 forms an inter-subunit hydrophobic contact with L120 indicating its involvement in macro-assembly formation. Mutational studies will further reveal the exact function associated with these residues. The remaining evolutionarily conserved A59 is buried with no solvent accessibility and forms a hydrophobic cluster with F23, M60, M61, L62 and V63. As expected, only hydrophobic residues occupy structurally equivalent positions of F23, L62 and V63 in other ferritins, but interestingly, charged residues reside in homologous ferritins at the M61 equivalent position of Mtb BfrB. For example, in human H-chain ferritin, the equivalent K68 (along with R76 and K146) is positioned in the internal wall of the cavity surrounding the negatively charged E131 and E134 residues that form the 3-fold channel. The presence of positive potential adjoining the entrance to the negative 3-fold channel is expected to enhance the effective size of the channel in directing cations to the interior of the protein [49]. In mycobacterial ferritins, the presence of conserved hydrophobic stretch (^59^AMMLVQ^64^) negates this analogous function associated with the stretch and in all probability plays a different role as compared to the postulated role in human H-chain ferritin.

The ConSurf analysis identifies P42, V53, E74, D79, D97, T101, W127, A151, F159, R162, V164, and D165 as the most hypervariable and rapidly evolving positions (Figure 6A). R162, in addition to forming an intra-subunit salt bridge (2.5 Å) with E163, also forms an inter-subunit hydrogen bond with N158 and a cation-pi interaction with F154 (Table 3). All these residues comprise the short E-helix that defines the 4-fold octahedral axis and plays a role in oligomerization and stability of the protein. Evolutionary conservation of these residues (E153, F154, N158 and R162) appears to be species specific as they are completely conserved in mycobacterial ferritins, but not in other ferritins. Similarly, P42 and V53 are unique to the species and display complete conservation indicating a significant purpose of these residues for ferritins belonging to mycobacterial species. Based on the regions of conserved residues in the listed ferritin homologues (with known structures) in Figure 3, a phylogenetic tree computed by ConSurf groups the mycobacterial ferritins, Bfrs and Dps as one cluster (Figure 6B) and eukaryotic ferritins as another. This supports the notion that the conservation/divergence in surfaces of different ferritin paralogues occurred amongst structurally-functionally critical residues that form these surfaces at the same rate presumably dictated by the need of the organism to perform different functions.

Ferritins show plasticity of the ferroxidase di-iron active sites. Subtle structural differences in the active-site ligands affect ferritin catalysis [56]. The environment of ferroxidase centres of representative ferritin members in Figure 7 clearly shows the similarities and differences existing amongst them. Comparison of the ferroxidase centre of Mtb BfrB with that of *E. coli* ferritin reveals the subtle differences: slightly altered conformations of E131 and E134 as compared to E126 and E129 respectively of *E. coli*, and flipped orientation of E54 in Mtb BfrB that is stabilized by formation of a salt bridge with R180. This later interaction is unique to mycobacterium species (Table 3). Further, H46 in *E. coli* has been substituted by Q51 in Mtb. As the side-chains of His and Gln are similar in both molecular size and ability to participate in hydrogen-bond as a proton donor/acceptor, this substitution is expected to make the least alteration on protein's functionality from physiochemical point of view. A study with bovine myoglobin (Mb) where a His that directly interacts with the Fe bound-ligand was mutated to Asn and Gln, revealed that though steric hindrance affects the ligand association to Mb, it is the strength of the hydrogen bond formed between the ligand and the residue that dictates the rate of ligand dissociation [57]. Consequently, modulation in the ligand dissociation rate is expected to affect the rate of oxidation at the di-iron centre and hence mineralization at the core in Mtb BfrB as compared to that of *E. coli* ferritin. Comparing the ferroxidase centre of Mtb BfrB with that of heme-containing Mtb BfrA shows that each iron in addition to being bridged by two carboxylate groups is coordinated by a His and a Glu residue in the latter, as opposed to Mtb BfrB, where only one metal site has a His ligand (Figure 7G and 7A). The location of ferroxidase centre of mycobacterial Dps proteins as reported by Roy et. al, is in a shallow groove at the dimer interface, enclosing not one but two di-iron sites [58]. Here, the metal binding residues are contributed by two subunits. Fe1 has five protein ligands and Fe2 is loosely coordinated by only one direct protein ligand and additional water contact (Figure 7H). Comparison of di-iron centres of 24mer ferritins, mycobacterial Bfrs and 12mer Dps proteins (Figure 7) emphasize the chemical and structural similarity among the family members and reinforce the plasticity of the active site.

Structure-function correlation in proteins is associated with electrostatic interactions and they have been shown in some cases to determine the thermodynamics and kinetics of macromolecular association [59]. At long range distances, the electrostatics can steer the incoming ligand to the active site whereas at short distances, they provide the specific local interactions for catalysis. A number of crystallographic studies on human, horse and mouse ferritins substantiates the role of the 3-fold channel in the entry of divalent cations into the interior of the protein [7], [60], [61]. Electrostatic potential energy calculations on these channels in mammalian H- and L-chain ferritins also espouse this observation [49], [62]. No negatively charged 3-fold channel is observed when electrostatic potential surfaces are compared in bacterial ferrritins, and a pore that is located ∼6 Å below the 3-fold channel entrance is proposed as a possible alternative route for iron uptake through the protein shell [4]. An electrostatic gradient is also observed through the negatively charged hydrophilic pores at the 3- fold axes of dodecameric ferritin-like Dps proteins that confirm the general picture that the electrostatics guide iron to the ferroxidase centre [19], [21].

Using the known crystal structures of the ferritins listed in Figure 3, electrostatic potential energy was computed for a single subunit as well as for the ferroxidase centre. Unfortunately, due to practical problems associated with performing calculations with large assembly, the electrostatic potentials of the whole quaternary structure of listed ferritins could not be compared. Nevertheless, PIPSA-generated cluster diagram for the subunits (Figure 10A) show *Pyrococcus furiosus* and soybean to be closest neighbours of Mtb BfrB while *Campylobacter jejuni* and *Helicobacter pylori* shows maximum similarlty when restricted to ferroxidase centre (Figure 10B). In both type of analysis the farthest homologue of Mtb BfrB is Bullfrog L-chain ferritin. Electrostatic potential was also calculated for complete assembly of Mtb BfrB so as to elucidate the functional properties of 3-fold, 4-fold channels and pores that are thought to provide pathways for the transfer of Fe(II) ions in and out of the protein. Surprisingly, the mouth of the 4-fold channel forming the inner cavity exhibits completely positive potential while that from outside displays negative potential (Figure 8). As 2 protons are released for each iron ion entering the mineral core [63], efficient proton removal from the site of mineralization is critical. It has been proposed that the large electrostatic field at the 4-fold channel of human H-chain ferritin may facilitate hydrogen ion exclusion from inside of the protein shell [62]. One can speculate that the 4-fold channel of Mtb BfrB plays the same role but this needs to be validated. Alternatively, the presence of an inter-subunit pi-cation interaction between R162 and F154 at the 4-fold channel (Figure 8 and Table 3), that appears to be a gate to the channel from the inner side, suggests in addition to subunit stabilization another function associated with these residues. F154 faces F159 from an adjacent subunit and a path consisting of tyrosine and phenylalanine residues (F159, Y35, F36, F48, F85 and Y29) leading from the internal cavity to the di-iron centre along the internal side of the helix bundle may constitute a route for electron transfer during iron uptake and release, similar to what has been proposed for the human H-chain ferritin, *Desulfovibrio desulfuricans* Bfr and Mtb BfrA [4], [38]. An intriguing interplay between F154 of one subunit and R148, E155 and R162 of another subunit (Table 3) further suggests stability and functional role for these residues. Anion-pi interactions are gaining significant recognition, and their pivotal role is advantageously exploited for anion transfer [64]. In order to attract a negative charge, the charge distribution of the pi system has to be reversed. This is achieved by placing a strong electron withdrawing group such as E155 along the pi system. F154, thereby, resulting in anions purportedly sliding along the electron-deficient pi clouds, again reinforce the above mentioned role in electron transfer. The presence of an anion in the vicinity of the pi system leads to a large redistribution of electron density and hence leads to an inductive stabilization [65]. The importance of noncovalent interactions involving aromatic systems and the interplay among them has been proposed to lead to strong synergic effects [66].

The inter- and intra-subunit interaction analysis of Mtb BfrB with other family representatives (Table 3) reveals that only few interactions such as the R69-D118 intra-subunit ion pair discussed above are conserved in all ferritin representatives. This indicates differences in the strength and types of interactions responsible for the folding, stability and functioning of a specific ferritin assembly. Different sets of conserved residues dictate stability of the assembly and channel/pore structures. Although they may vary only subtly but the variation is difficult to decipher or correlate just based on sequence-structure-function comparisons. All three types of ferritins discussed (ferritin, Bfr and Dps) store iron, but also have more specialized functions in iron detoxfication, redox stress response or DNA protection. The precise roles of these ferritins vary depending on the organism and cell type [25]. The interplay between different ferritins co-existing in the same cell and the diversity of their physiological role is the topic of ongoing research in many laboratories.

In conclusion, we have determined the three-dimensional crystal structure of Mtb BfrB. The structure gives insights into the residues playing important roles in the structural and functional integrity of the protein and suggests further mutagenesis experiments to probe the importance of these residues. Comparative analysis with representative homologues belonging to all realms reinforce the fact that ferritins are dynamic protein cages and lack conservation vis-à-vis residues defining gated channels/pores that control flow of iron, oxidants and reductants. In addition, the crystallographic data as well as the biochemical studies implicate the importance of the extended C-terminal region in the iron entry from the three-fold channels to the ferroxidase centre and making iron more readily accessible for the oxidation. Although the basic mechanisms of iron entry/exit and oxidation are known, the specificities associated with a particular ferritin in context to cellular needs cannot be explained by comparative studies alone. Much needs to be learned before specific conclusions can be drawn and as such ferritin remains the subject of active research.

## Materials and Methods

### Materials, bacterial strains and growth conditions

All reagents were obtained from Sigma-Aldrich Inc. (St. Louis, MO, USA). Sephacryl-300 resin was procured from GE healthcare (Uppsala, Sweden). *E. coli* BL21 (λDE3) cells were grown in Luria Bertani (LB) broth at 37°C with constant shaking at 200 rpm. Wherever appropriate, ampicillin was added at a concentration of 50 µg/ml.

### Cloning and expression of *bfrb* gene

The gene encoding BfrB (Rv3841) was PCR amplified using *M. tuberculosis* H37Rv genomic DNA as template. The primers were designed based on the sequence available from the EMBL/GenBank. 5′gaattcggatccgctagcacagaatacgaagggcctaag 3′ containing *Bam*HI and *Nhe*I sites was used as the forward primer and 5′gaattcaagctttcactagaggcggcccccggcagc 3′containing *Hind*III site was used as reverse primer for PCR amplification. The PCR amplicon of the *bfrb* gene was cloned in to the pET21c vector at *Nhe*I and *Hind*III sites and the legitimacy of the construct was verified by DNA sequencing. For expression studies, *E. coli* BL21 (λDE3) cells transformed with recombinant plasmid were used to inoculate LB medium. The cells were grown at 37°C (200 rpm) to mid-logarithmic phase followed by expression of the recombinant protein by induction with 1 mM isoproryl-1-thio-β-D-galactopyranoside (IPTG) for 3 hours at the same temperature.

The amplification of the truncated bfrb gene (encoding residues 1–167) was carried out by using the above mentioned forward primer and 5′gaattcaagcttttactacgccacatccacttcacg 3′ containing the *Hind*III site as the reverse primer. The selected clones after verification of the sequence were subjected to expression studies.

### Purification of BfrB and its truncated mutant

Recombinant proteins were synthesized in 2 litre LB broth as described in the above section. Cells were harvested by centrifugation at 4°C, 6000 g for 10 minutes. Harvested cells were re-suspended in 25 ml of re-suspension buffer (20 mM Tris.Cl, 100 mM NaCl, 1 mM PMSF, 2 mM β-mercaptoethanol pH 8.0) and lysed by using French Press. The resulting cell lysate was centrifuged at 12,000 g for 1 hour at 4°C and subjected to 0–25% ammonium sulfate precipitation. The precipitate after solublizing in re-suspension buffer was further purified by size-exclusion chromatography. A Sephacryl S-300 (2.5 cm×92 cm) column pre-equilibrated with 20 mM Tris.Cl, 100 mM NaCl pH 8.0 was loaded with 5 ml of sample (containing ∼35 mg of protein) and run at a flow rate of 0.5 ml/min. Fractions of 5 ml volume were collected and analyzed on a 12.5% SDS-PAG for purity.

### Crystallization of BfrB

The fractions containing pure protein were pooled and concentrated to 11 mg/ml for crystallization (using Amicon stirred cell with 100 kDa cutoff membrane). PACT suite (QIAGEN India Pvt. Ltd., Middle Circle, Connaught Place, New Delhi, India) was used for initial screening. Best optimized crystals were grown by vapor diffusion at 20°C in drops containing 1 µl of 11 mg/ml of recombinant BfrB mixed and equilibrated with equal volume of reservoir buffer containing 20% PEG3350 in 0.1 M Tris-HCl pH 8.5.

### Data Collection, Structure solution and refinement

Diffraction data were collected from a single crystal flash-cooled at 120 K in a stream of N_2_ gas. The cryoprotection conditions consisted of equal volumes of the mother liquor and 60% PEG4000. Data were recorded on a MAR image plate detector (345 mm diameter circle, 0.15 mm pixels) using a copper-target rotating anode. Results are summarized in Table 1. The lattice was autoindexed with LABELIT [67], followed by integration with MOSFLM and scaling with SCALA, both components of the CCP4 program suite [68]. The crystal was initially rotated through a 200° wedge, sufficient to produce a complete dataset. However, interference from overlapping lattices limited data integration to the first 104°, producing data that were partly incomplete (Table 1). However, it was thought that the 24-fold non-crystallographic symmetry present in the asymmetric unit would compensate for any negative impact from the data's incompleteness. Crystallographic phases were obtained by molecular replacement, with a search model consisting of a complete 24-chain shell derived from the archaeal *Thermotoga maritima* ferritin (PDB code: 1vlg). Partial complexes consisting of one and six chains were also evaluated as search models, but with negative results. To construct the search model, multiple ferritin protein sequences from eubacteria, archaea and eukaryota were aligned with the program ClustalW [69], and the sequence alignment was compared to known crystallographic structures from the PDB (Table 2). The use of eukaryotic models was ruled out because these sequences contain a one amino acid insertion within helix D relative to BfrB, while ferritin sequences from the eubacterial and archaeal groups maintain a constant-length core (156 residues) extending from helix A through helix E (Figure 3). Structure 1VLG was selected since it has a high sequence identity to BfrB and best limiting resolution (2.0 Å) of all candidates considered. With a 24-chain search model consisting of 1VLG residues K7 through Q162 (truncated to alanine or glycine for sidechain positions differing from BfrB), a clear solution was obtained with the phenix.automr module of PHENIX [70]. Refinement was carried out with the phenix.refine module in conjunction with iterative manual model building in COOT [71]. Later it was realized that the search model differed from the final BfrB structure in both the length of the linker joining helices D and E, and also the orientation of helix E; therefore the phasing power of the model was due to its similarity to helices A through D only.

### Structure based multiple sequence alignment

Structure based multiple sequence alignment of Mtb BfrB with representative ferritin structures was generated with DALI [44] and further modified by inspecting the pair wise superimposition manually.

### Site-specific conservation pattern among ferritin family members

The degree of conservation of each amino acid in the ferritin family was analyzed by estimating site-specific evolutionary rates using the ML (maximum likelihood) approach of the ConSurf server [72]. The analysis was conducted by providing the structure of Mtb BfrB and multiple sequence alignment (Figure 3) as input to the program.

### Analysis of surface electrostatic potentials

Molecular potential of the structures were computed and analyzed by webPIPSA [73]. Electrostatic potentials were computed using UHBD (University of Houston Brownian Dynamics) assuming an ionic strength of 100 mM, a temperature of 300 K and treating the protein as a low dielectric with partial atomic charges embedded in a homogeneous high dielectric continuum representing the solvent. Superimposed set of coordinate file of single subunit of each ferritin was submitted to the server so as to ensure that whole proteins as well as ferroxidase centres at equivalent locations were compared. The result of webPIPSA is displayed as a color-coded distance matrix. These distances are used to group the proteins according to the relations between their electrostatic potentials in a cluster dendogram.

### Circular Dichroism of BfrB and Truncated BfrB

Far-UV CD spectra were recorded on J-815 spectropolarimeter (JASCO Corporation, Hachioji-shi, Tokyo, Japan). An average of 3 scans was taken and the spectra were obtained at an interval of 0.1 nm with a scanning speed of 50 nm/min by using a 1 mm path length quartz cuvette. A protein concentration of 0.05 mg/ml in 10 mM sodium phosphate, pH 8.0 was employed. Spectra were recorded at various temperatures maintained through an attached water bath with 5 minutes incubation at the desired temperatures.

### Kinetic studies of Iron oxidation and Release

To a protein solution of 0.25 µM, 125 µM of Ammonium ferrous sulphate freshly prepared in 0.015 N HCl was added in 0.1 M HEPES, pH 6.5. The iron oxidation was monitored by increase in the optical density at 310 nm, which specifically measures Fe(III) ions and the kinetic measurements were recorded from the time of addition of iron to the protein. For iron release experiments, 0.25 µM protein was mineralized with 125 µM ammonium ferrous sulphate in 0.1 M HEPES, pH 6.5, followed by incubation at room temperature for 2 hours. Iron release was initiated by the addition of 1 mM ferrozine reagent prepared in 0.1 M HEPES pH 7.0 and 250 mM sodium ascorbate. The quantity of released iron was measured kinetically by monitoring the absorbance of the Fe(II)-Ferrozine complex at 570 nm.
